# Biosimilar structural comparability assessment by NMR: from small proteins to monoclonal antibodies

**DOI:** 10.1038/srep32201

**Published:** 2016-08-31

**Authors:** Boštjan Japelj, Gregor Ilc, Jaka Marušič, Jure Senčar, Drago Kuzman, Janez Plavec

**Affiliations:** 1 Protein Biophysics and Bioinformatics Department, Sandoz Biopharmaceuticals, Kolodvorska 27, SI-1234 Mengeš Slovenia; 2 Slovenian NMR Centre, National Institute of Chemistry, Hajdrihova 19, SI-1000 Ljubljana, Slovenia; 3 EN-FIST Centre of Excellence, Trg Osvobodilne fronte 13, SI-1001, Ljubljana, Slovenia; 4 Faculty of Chemistry and Chemical Technology University of Ljubljana, Večna pot 113, SI-1000 Ljubljana, Slovenia

## Abstract

Biosimilar drug products must have a demonstrated similarity with respect to the reference product’s molecules in order to ensure both the effectiveness of the drug and the patients’ safety. In this paper the fusion framework of a highly sensitive NMR fingerprinting approach for conformational changes and mathematically-based biosimilarity metrics is introduced. The final goal is to translate the complex spectral information into biosimilarity scores, which are then used to estimate the degree of similarity between the biosimilar and the reference product. The proposed method was successfully applied to a small protein, i.e., filgrastim (neutropenia treatment), which is the first biosimilar approved in the United States, and a relatively large protein, i.e., monoclonal antibody rituximab (lymphoma treatment). This innovative approach introduces a new level of sensitivity to structural changes that are induced by, e.g., a small pH shift or other changes in the protein formulation.

The patents for the first generation of approved biological drugs have either already expired or are about to expire in the near future, opening the market for biosimilars^1^. Biosimilars are expected to reduce the costs of treatment and thus allow greater access to biologic therapies for patients^2^. Unlike small molecules, which are produced by chemical syntheses, biological drugs are produced through complex processes involving living cells^3^. Replicating protein molecules is a much more demanding task due to their structural complexity, intricate manufacturing processes (cell lines, raw materials and equipment) and the potential safety risks. This is particularly relevant as the immunogenicity of biological drugs as a safety issue has received considerable attention in recent years, confirming the need for comprehensive testing prior to approval and an extended period of post-marketing surveillance^1^,^4^,^5^,^6^.

A comparison of protein molecules, i.e., the biosimilar drug with the reference product, is a challenging task that involves an extensive physicochemical and functional characterization as well as animal toxicity, human pharmacokinetics/pharmacodynamics, immunogenicity, and clinical safety and effectiveness using a stepwise approach^7^. There are several methods available to characterize the high-order structure of a protein, i.e., the physicochemical (e.g., NMR spectroscopy, X-ray crystallography, electron microscopy, microcalorimetry, hydrogen/deuterium exchange with mass spectrometry etc.) and the functional assays^8^.

Since the three-dimensional structure of a protein is an important factor in its biological function, any differences in the high-order structure between a proposed biosimilar drug and the reference product must be evaluated in terms of any potential effects on the protein’s function. Differences in the protein’s structure could lead to a changed activity and undesired side effects in patients, and thus extreme caution is required.

A limited number of studies were so far published where authors used NMR fingerprint spectra to study higher order protein structure (HOS) and compare it to the reference product^9^,^10^,^11^,^12^,^13^. Aubin Y. *et al*. explored the sensitivity of the NMR spectroscopy to structural changes induced by experimental conditions such as changes in pH, ionic strength, buffers, excipients and residue mutations. Ghasriani H. *et al*. addressed the precision and robustness of the 2D-NMR for structure assessment in an inter-laboratory comparative study.

The goal of biosmilar development is to be highly similar to the reference product. In this paper we present a new, NMR-bioinformatics framework that is able to systematically evaluate the high-order structural similarity between a biosimilar drug and the reference product. The framework starts by recording the homo- and hetero-nuclear, multi-dimensional NMR spectra of proteins under carefully controlled solution conditions. The NMR spectral fingerprints that sample the structure at different levels are then compared using mathematical based metrics that can be divided into three main categories: a peak-to-peak comparison, a global comparison and an image analysis. This approach is an extension of the classical qualitative inspection of spectral overlays, which are a powerful comparison tool, but are also prone to subjective human interpretation. In contrast, our data-driven approach provides objectivity, since the criteria are defined prior to the analysis. The study was successfully performed for a relatively small protein (~19 kDa), i.e., a granulocyte colony stimulating factor (indicated for the treatment of neutropenia), and a relatively large protein (~145 kDa), i.e., monoclonal antibody rituximab (used for the treatment of non–Hodgkin lymphoma and chronic lymphocytic leukemia) (Fig. 1)^14^,^15^,^16^,^17^. Based on the results obtained for the small and the large proteins, we showed that the described NMR-bioinformatics framework is an essential tool that contributes to the completeness of the totality of evidence for demonstrating similarity to the reference product.

## Results

### NMR spectroscopy

The similarity study was performed on two different proteins: an 18.8 kDa protein filgrastim (G-CSF, granulocyte colony-stimulating factor, reference product Amgen trade name Neupogen and Sandoz trade name Zarxio, which is the first biosimilar approved in the US) and 144.5 kDa monoclonal antibody rituximab (reference product Roche trade name MabThera and Sandoz biosimilar rituximab). From this point forward originator filgrastim will be used for Neupogen, biosimilar filgrastim for Zarxio, originator rituximab for MabThera and biosimilar rituximab for Sandoz biosimilar rituximab. The similarity was evaluated using qualitative NMR spectral overlays and quantitative bioinformatics comparability methods, the purpose of which was to convert the complex spectral information into similarity scores.

**Figure 2:**
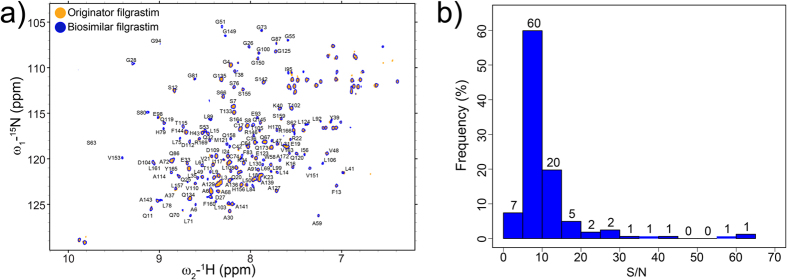
Comparative analysis of ^1^H-^15^N HSQC spectra of filgrastim reference product (orange) and biosimilar filgrastim product (blue). (**a**) Overlay of the amide fingerprint spectra. (**b**) Histogram of S/N ratios of the HN cross-peaks for the originator filgrastim US sample.

The ^1^H-^15^N HSQC and ^1^H-^1^H NOESY NMR spectra were acquired for the biosimilar and originator filgrastim products to obtain the amide fingerprints and the through space dipolar correlations. A complete cross-peak overlay of the ^1^H-^15^N HSQC spectra indicated the highest level of similarity for the compared proteins and their three-dimensional structures (Fig. 2a). An acquisition time of 31.5 h was used to achieve a favourable signal-to-noise (S/N) ratio on an isotopically (0.4% ^15^N natural abundance) unlabelled sample resulting in 100% signal coverage (Fig. 2b). Most of the observed cross-peaks, i.e., 60% had S/N ratios from 5–10, 20% had S/N ratios between 10 and 15, and only two cross-peaks exhibited a S/N ratio of 4; however, this was still sufficient for an unequivocal identification.

The strong influence of pH on the ^1^H and ^15^N resonance positions of the V48, G73, S76, S80, L103, D104, A127, S142 and L161 cross-peaks was observed when the pH was increased from 4.0 to 4.4 in the originator and biosimilar filgrastim drug-product formulations, respectively. In order to systematically demonstrate the sensitivity of the method to pH changes, the biosimilar filgrastim samples were prepared in three different formulations, with the pH values of 3.0, 4.0 and 4.4. The ^1^H-^15^N HSQC spectra demonstrated a clear response to the pH changes (Supplementary Fig. S2).

## Mathematically based metrics

### Peak-to-peak comparison

**Figure 3:**
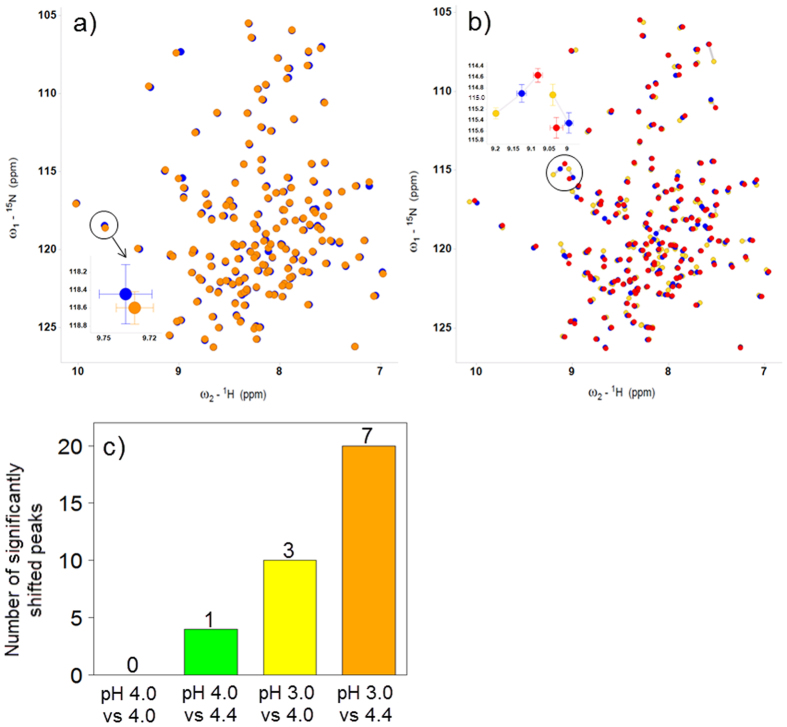
Statistical analysis of ^1^H-^15^N HSQC spectra of biosimilar filgrastim (blue) and originator filgrastim US (orange). (**a**) Overlay of the cross-peak positions in the pH 4.0 formulation for the t-test analogue evaluation. (**b**) Overlay of the biosimilar filgrastim at three different pH values: 3.0 (yellow), 4.0 (blue) and 4.4 (red). (**c**) Number of significantly shifted peaks of pairwise compared spectra in the pH 4.0, 4.4 and 3.0 formulations (0 peaks for pH 4.0 vs pH 4.0, 1 peak for pH 4.0 vs pH 4.4, 3 peaks for pH 3.0 vs pH 4.0 and 7 peaks for pH 3.0 vs pH 4.4 formulation).

Three biosimilar filgrastim drug-product batches were compared to the originator filgrastim EU and US reference product using a t-test analogue separately for both formulations, pH 4.0 and pH 4.4, as described in the Methods section. The centres of the individual cross-peaks in the ^1^H and ^15^N spectra served as location estimates, whereas the peak-width reflected the variation estimate. Chemical-shift changes in the ^1^H-^15^N HSQC spectra of two samples were identified as significant if the p-value was less than 0.05 and the S/N ratios of both cross-peaks were larger than 3. No significant peak shifts were identified when the samples of biosimilar and originator filgrastim were compared pairwise (Fig. 3a), with the exception of the E45 cross-peak, which could not be resolved from the noise level.

The sensitivity of the t-test analogue approach was further demonstrated by its ability to detect small, pH-induced structural changes through an analysis of the ^1^H-^15^N HSQC spectra at different pH levels: 3.0, 4.0 and 4.4 (Fig. 3b). A pairwise comparison of the spectra using the t-test analogue approach revealed that such a statistical analysis was able to detect significant structure-related shifts induced by a pH change as small as 0.4 units. The overlay of the biosimilar filgrastim batch materials formulated at pH 3.0, 4.0 and 4.4 are shown in Fig. 3b. The pH change from 4.0 to 4.4 resulted in 1 significant chemical shift; the pH change from 4.0 to 3.0 resulted in 3; and the pH change from 3.0 to 4.4 resulted in 7 significant chemical shifts using Benjamini-Hochberg correction for multiple tests^18^,^19^. The number of significant shifts without correction for multiple testing was 0 for the same pH, 4 for Δ*pH* = 0.4 units, 10 for Δ*pH* = 1.0 and 20 Δ*pH* = 1.4 units. These results clearly demonstrated sufficient sensitivity to detect minor structural changes in the protein through an analysis of the ^1^H-^15^N HSQC spectra (Fig. 3c). The most pronounced pH-induced chemical-shift changes (Δδ) were observed for the cross-peaks corresponding to C42, S80, H97, D104, D112, S142, and L161.

### Summarization-score-based global comparison

#### Principal component analysis

**Figure 4:**
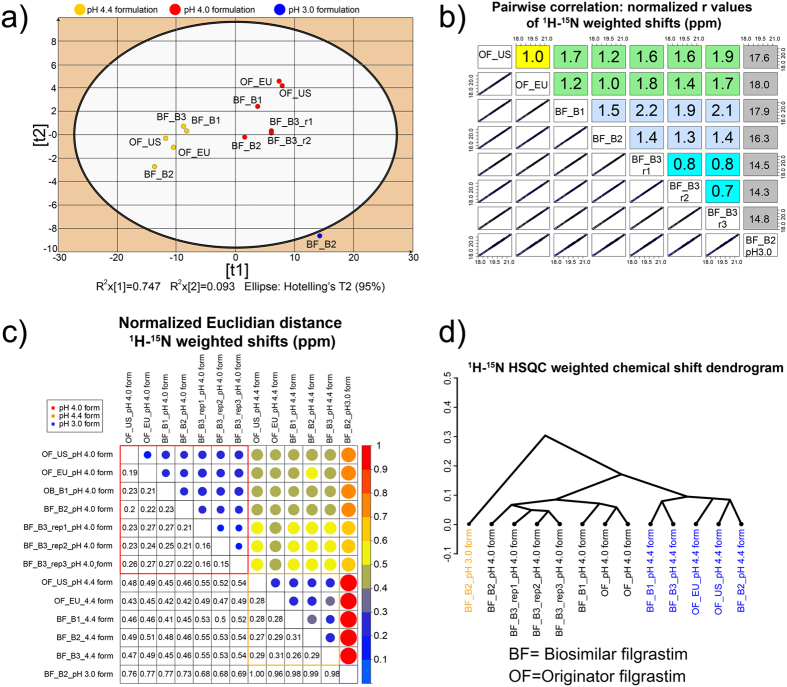
Similarity between biosimilar (BF) and originator filgrastim (OF) spectra in the pH 3.0, 4.0 and 4.4 formulation. (**a**) PCA scores showing the high similarity of the biosimilar filgrastim batches to the reference batches. Coloured groups (yellow, red, blue) represent different formulations. (**b**) Pairwise ^1^H and ^15^N weighted chemical-shift correlation coefficients between the originator and biosimilar filgrastim batches in pH 4.0 and pH 3.0 formulation. The yellow rectangle shows the scaled r value for US and EU originator, the green rectangles shows the comparison between OF and BF in the pH 4.0 formulation, the cyan rectangles comparison between the 3 BF batches and blue comparison between the three replicates of the same sample. The grey rectangles shows the scaled r values for comparison between pH 4.0 and 3.0 formulations. (**c**) Normalized Euclidean distance metrics for ^1^H and ^15^N weighted chemical-shift shifts in pH 4.0, pH 4.4 and pH 3.0 formulation. The normalized distances are shown as circles with a scaled diameter and colour in the upper triangular matrix, whereas the corresponding values shown as numbers are mirrored through the diagonal to the lower triangular matrix. (**d**) Hierarchical clustering of the ^1^H and ^15^N chemical shifts.

A principal component analysis (PCA) was performed on the ^1^H-^15^N HSQC spectra of 13 samples using 162 ^1^H and ^15^N chemical shifts as the X-variables^20^,^21^. The PCA model reduced the variability in the X data by projecting the original point swarm in a multivariate, high-dimensional space to a hyperplane of 2 principal components (t1 and t2 in Fig. 4a), best describing the variability in the X-data (the 84% data variation was accounted for by t1 and t2). The scores t1 and t2 were clearly separated into three distinct groups with respect to the formulation pH value. From the group separation it is clear that the distinct formulations are unequivocally differentiated using the PCA of the ^1^H and ^15^N chemical shifts. The originator US and EU and the biosimilar batches 1 to 3 are comparable within two separate formulations, whereas the batch-2 in the pH 3 buffer is well separated, according to the circumference of the Hotelling’s T2 ellipse at the 95% confidence level. These results show that the batches of the biosimilar filgrastim drug product are comparable to the originator filgrastim US and EU product batches under the same conditions used in the formulations.

#### Correlation method

The pairwise similarity between the spectra was measured using the Pearson correlation coefficient (r)^22^. The degree and direction of the linearity was calculated between the chemical shifts and the peak intensities extracted from the ^1^H-^15^N HSQC spectra (Fig. 4b)^23^,^24^. The large number of weighted ^1^H and ^15^N chemical shifts and the relatively small Δδ values resulted in r^2^ values ranging from 0.995 to 1.000. The r^2^ values were transformed so that the reference product r^2^ value had a value of 1.0, whereas all the other r^2^ values were scaled using the reference-product scaling factor. The results (Fig. 4b) confirm the excellent agreement between the originator filgrastim US and EU reference product and the three replicates of biosimilar filgrastim, whereas the pH 3.0 formulation deviated from linearity as a result of the pH-induced chemical shifts.

#### Distance metrics

The similarity between the ^1^H-^15^N HSQC of the biosimilar filgrastim drug batches and the two originator filgrastim US and EU product batches spectra was measured using the Euclidean distance. The analysis showed that the distances between the biosimilar filgrastim drug product batches are comparable to the distances between the two originator filgrastim US and EU drug product batches (Fig. 4c). To control the sensitivity of the analysis the samples were prepared in different buffer solutions and the analysed distances for those samples were much greater with respect to the consistent samples (the red, orange, yellow and green vs. blue circles in Fig. 4c).

#### Hierarchical cluster analysis

An agglomerative hierarchical cluster analysis was used to group the ^1^H and ^15^N chemical shifts into clusters with respect to the similarity between the samples^25^. The results of the clustering were visualized as a dendrogram in Fig. 4d, which shows the sequence of the cluster fusion and the distance at which each fusion took place. The results confirmed the similarity between the biosimilar and the originator filgrastim and showed a strong similarity within the same buffer and small differences between the different buffers.

#### Tolerance interval approach

The main assumption of the tolerance interval peak-shift approach is that the peak is significantly shifted if the difference is outside the tolerance interval of the reference product.

**Figure 5:**
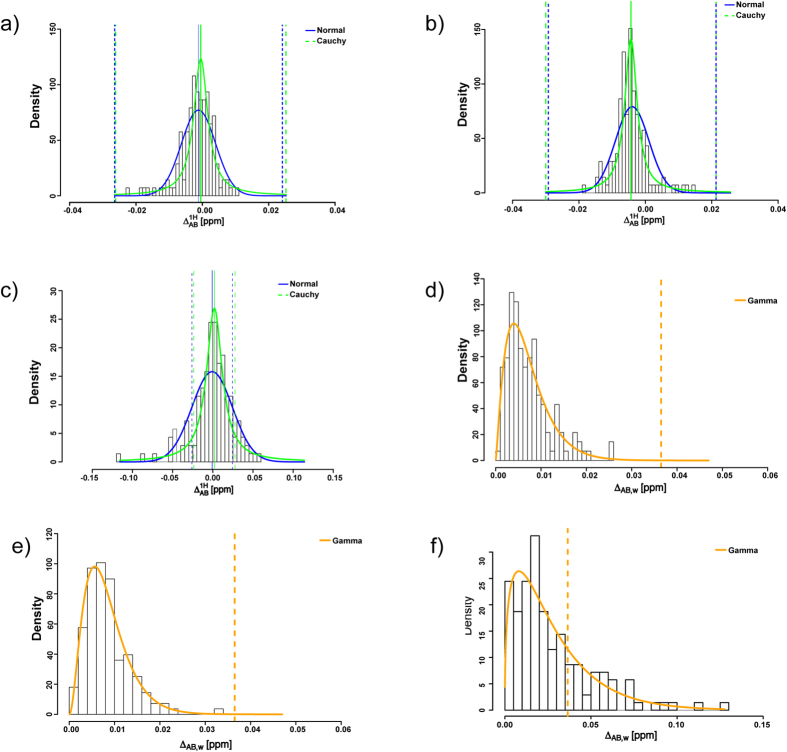
Histograms of peak-shift distances between the biosimilar and originator filgrastim products. Tolerance intervals for the normal, Cauchy and Gamma distributions are shown as blue, green and orange dashed lines, respectively. (**a**) ^1^H chemical shift difference histogram of originator filgrastim US and EU batch in the pH 4.0 formulation. (**b**) ^1^H chemical shift difference histogram of biosimilar filgrastim batch 1 and originator US batch in the pH 4.0 formulation. (**c**) biosimilar filgrastim batch 2 in pH 4.0 and pH 3.0 formulation. (**d**) Gamma distribution of the weighted chemical shift differences of the originator filgrastim US and EU batch in the pH 4.0 formulation. (**e**) biosimilar filgrastim batch 1 and originator US batch in the pH 4.0 formulation and (**f**) biosimilar filgrastim batch 1 in pH 4.0 and pH 3.0 formulation.

The ^1^H-^15^N HSQC histograms for the pairwise chemical shift differences with a tolerance interval for the Cauchy, Normal and Gamma distributions are shown in Fig. 5. The Fig. 5a,d show a similarity between the originator filgrastim US and EU batches, Fig. 5b,e show a similarity between the biosimilar and the originator US filgrastim. The sensitivity of this method is demonstrated in Fig. 5c,f, which show histograms of the biosimilar filgrastim batch in two different formulations: pH 4.0 and 3.0. All the originator filgrastim US and EU pairwise differences fell within the tolerance intervals for the Cauchy, Normal and Gamma distributions at a confidence level of 0.95 and P (coverage) values of 0.90, 0.99 and 0.99, which were determined for the reference product samples, respectively. With a pH change of 1.0 unit, 24%, 25% and 26% of the peaks fell outside the tolerance interval for the Cauchy, Normal and Gamma distributions, respectively. The tolerance-interval approach results for the biosimilar rituximab are shown in Supplementary Fig. S3.

### Image-difference analysis

**Figure 6:**
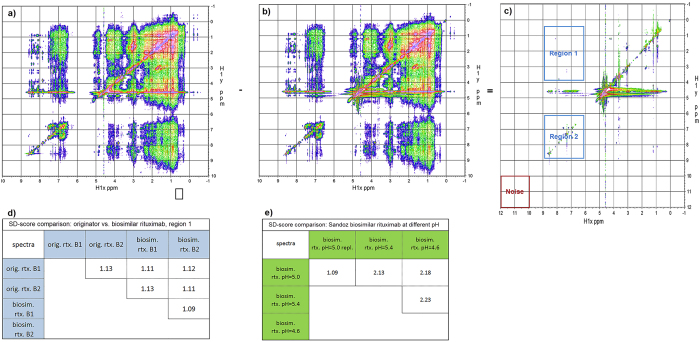
NOESY spectra of the full size monoclonal antibody rituximab (rtx. biosimilar and the reference product) and image difference analysis. (**a**) Processed NOESY spectrum of biosimilar rituximab batch 1, (**b**) of originator rituximab reference product sample and (**c**) difference spectrum with region 1 (aliphatic) and 2 (amide-aromatic) and region for the noise level determination. (**d**) Spectral difference scores for the comparison of the reference and biosimilar rituximab region 1. (**e**) Spectral difference scores for the comparison of the biosimilar rituximab at three different pH values.

The pairwise spectral difference was used to evaluate the image-difference-based similarity between the biosimilar and originator rituximab. Two regions of interest were defined in the NOESY spectrum, one in the amide-aromatic region (H_N_-H_ar_) and the other in the amide-aliphatic region (H_N_-H_β_, H_N_-H_γ_ and H_N_-H_δ_) (Fig. 6). The results were based on the difference (Fig. 6c) between two individual NOESY spectra (e.g., the biosimilar (Fig. 6a) and originator rituximab (Fig. 6b). Spectral-difference pairwise scores close to 1 represent a high similarity between the NOESY spectra of interest (the biosimilar vs. originator rituximab spectral-difference score was 1.13 (Fig. 6d). In order to demonstrate the sensitivity of the approach, one of the Sandoz biosimilar rituximab batches was prepared at pH values of 5.0, 4.6 and 5.4. The region-1 spectral-difference scores for the pairwise comparison increased from 1.09 for the replicates to 2.18 and 2.13 after the pH change of 0.4 units (Fig. 6e).

## Discussion

**Figure 7:**
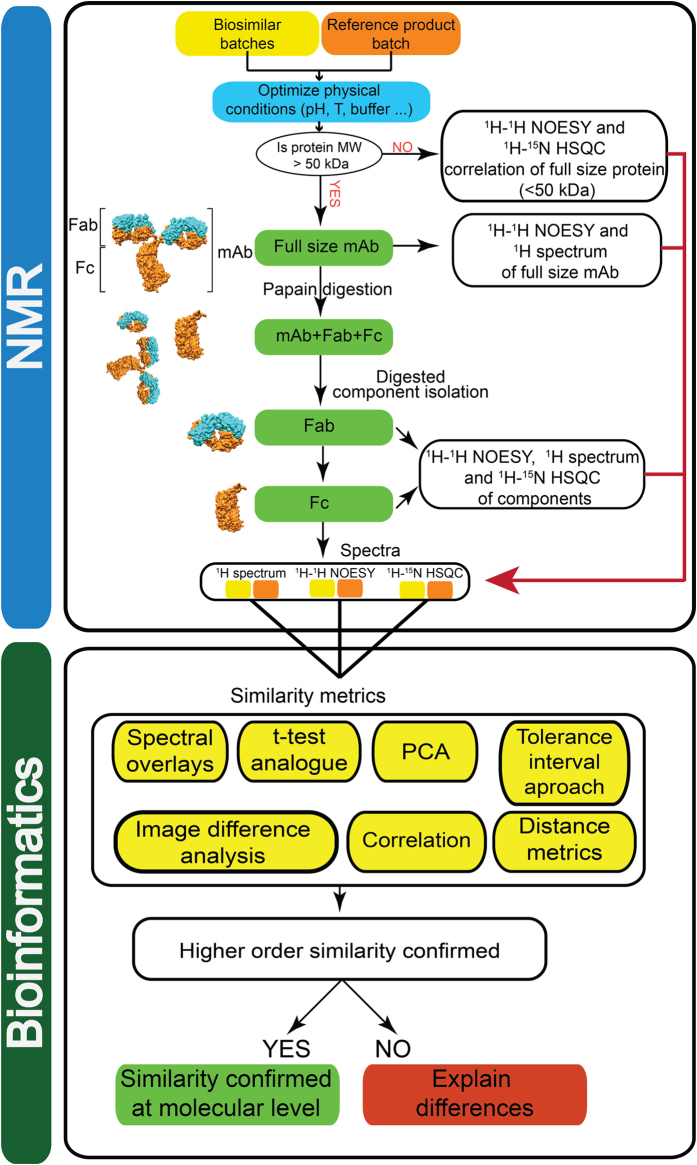
NMR fingerprint-bioinformatics workflow.

The main objective of our approach was to introduce a comparability framework that combined the use of NMR, to study the higher-order protein structure, and mathematically based metrics, to translate the complex spectral information into simplified similarity scores. The spectral fingerprints obtained using NMR are as unique for a specific protein as human fingerprints are for an individual person. The obtained spectra provide an insight into the biochemical structure, the integrity and the spatial structure of the biological drugs. It is important to emphasize that the flexible parts, which in principle give the strongest NMR signal, especially for larger proteins (e.g., rituximab), often play an important role in protein-protein interactions^26^,^27^. The proposed comparability framework (Fig. 7) can be roughly divided into the NMR part and the mathematically-based metrics (bioinformatics evaluation) that serves to analyse the results.

The NMR part starts with sample preparation, which has to be optimized in order to obtain a high-quality NMR spectrum. Ideally the proteins should be compared in the final drug product formulation. The formulation however usually contains excipients to stabilize the protein (e.g. Tween 80, sorbitol etc.) which can increase the viscosity of the sample resulting in broader signals. In the case of small proteins such as filgrastim this is not so critical whereas in the case of mAb an optimization of the sample conditions would result in higher signal coverage. In some cases the biosimilar and the reference product formulations are not the same (e.g. the Zarxio biosimilar and the Neupogen reference filgrastim formulations have pH of 4.4 and 4.0, respectively). In such cases it is important to perform the NMR comparability study in the same conditions, i.e compare the protein molecules in the same environment. The ^1^H-^15^N HSQC amide fingerprint and the NOESY spectra were used in our study. The experimental part is relatively simple for small proteins (e.g., filgrastim), but the complexity increases with the molecular size of the proteins and is difficult to obtain high signal coverage in spectra^28^,^29^,^30^. For the rituximab samples in the proposed approach proteolytic enzymes (e.g. papain) were used to digest the protein into Fab and Fc fragments that were isolated using preparative chromatography. Smaller fragments allowed us to perform the NMR analysis without any specific isotope-labelling techniques used to minimize the proton relaxation (normally the deuteration of the protein is used for this). Unlike filgrastim ^1^H-^15^N HSQC spectra where the signal coverage was 100%, the coverage of the rituximab Fab and Fc fragments was ~9% and ~7% respectively at signal-to noise threshold of 5^31^,^32^. Using this threshold 35 backbone and 20 sidechain signals out of the expected 411 signals (excluding Pro) were observed for Fab fragment in the ^1^H-^15^N HSQC spectra. With the lower S/N threshold 49 signals were observed (approx. 12% coverage). 13 backbone and 18 sidechain signals were observed above S/N of 5.0 for the Fc fragment (~7% coverage). Higher signal coverage could be achieved using techniques such as fast pulsing (e.g. BEST-HSQC, SOFAST-HMQC), non-uniform sampling and processing or ultrafast NMR methods using dynamic nuclear polarization^33^,^34^,^35^,^36^,^37^. Another alternative to ^1^H-^15^N HSQC spectra which could improve spectral coverage are ^1^H-^13^C HSQC spectra of the methyl groups^28^. The methyl groups which are present in six aminoacids (Ala, Ile, Leu, Met, Val, Thr) give rise to signals in a region of the spectrum which is not overlapping with other aliphatic resonances and thus serve as reliable reporters of correct protein folding. In addition the further benefits of the ^1^H-^13^C HSQC spectra include greater natural abundance of the ^13^C isotope (1.1%) compared ^15^N (0.4%) and better relaxation behaviour due to the free rotation of the methyl group. ^1^H-^13^C HSQC spectra however seem to be less sensitive to pH induced conformational changes than ^1^H-^15^N HSQC spectra and may be affected by the interference from excipients (e.g. sorbitol, polysorbate 80) and were thus not used as a primary fingerprint spectra for evaluation of HOS similarity^11^. A ^1^H-^13^C HSQC spectrum of biosimilar rituximab, pH 6.5 is included in Supplementary Fig. S4. It is important to note that the use of NOESY spectra, which have much higher sensitivity compared to ^1^H-^15^N HSQC spectra at natural isotopic abundance, are limited by the poor dispersion of the ^1^H resonances. Protein ^1^H backbone amide resonances are typically observed in the spectral range from 6.5 to 10 ppm compared to ^15^N resonances which are typically in the range from 100 to 130 ppm, i.e. an order of magnitude larger spectral range.

To increase the sensitivity of the spectra the experimental conditions, such as temperature, pH and protein concentration, had to be optimized. We were able to demonstrate that a pH change of 0.4, 1.0 or 1.4 pH units led to significant changes in the chemical shift positions. Similar observations were made before by Pujato and Panjwani^38^,^39^, confirming the high sensitivity of the NMR method with respect to detecting structural changes^40^.

The NMR experimental part resulted in high-quality, one-dimensional proton spectra and two-dimensional, ^1^H-^15^N HSQC and NOESY spectra. Such spectra are usually compared as simple overlays, followed by a visual inspection. Despite the fact that the human brain excels in detecting patterns and subtle differences in patterns, this ability varies from person to person. In order to lay down a strong mathematical basis for the similarity comparison, several techniques were used that can be roughly divided into three classes: a peak-to-peak comparison, a summarization score based on a global comparison and an image analysis. It needs to be emphasized that all bioinformatics methods can be used for small and large proteins, i.e., the methods used for filgrastim could be applied to rituximab and vice versa.

Several approaches for spectral fingerprint comparison were described in the literature. Zuperl *et al*. used two chemometrics approaches to compare 2D NOESY spectra^41^. The first method compared all the peaks in a selected region and determined the percentage of concurring peaks whereas the second was based on the sequential nearest neighbours to evaluate parts of the NOESY spectra. Amezcua *et al*. measured similarity between two samples using the correlation coefficient derived from linear regression analysis of binned NMR spectra^23^. Ghasriani *et al*. used combined chemical shift difference analysis (CCSD) and principal component analysis approach^9^. In addition to already described methods we introduced new methods for NMR comparability assessment such as t-test analogue, tolerance interval approach, distance metrics, hierarchical clustering and image difference analysis to derive SD-scores.

The peak-to-peak comparison method includes the t-test analogue, which performs multiple t-tests for each signal observed in the pair of spectra. The applicability of this method was shown for the filgrastim protein using data from the ^1^H-^15^N HSQC spectra, but in principle other types of spectra, such as ^1^H-^13^C HSQC, could also be used. The t-test analogue uses an idea from the sample mean comparison, where the Euclidean distance between two individual peaks is scaled by their width. None of the peaks was significantly shifted using the predefined criteria when the biosimilar protein was compared to the reference product in the same buffer showing a high similarity between the biosimilar filgrastim and the originator reference product.

The second set of bioinformatics methods summarized the NMR spectral data to scores that were in turn compared. These methods included the PCA, the correlation coefficient, the Euclidean distance, the hierarchical cluster analysis and the tolerance interval approach. The least sensitive of these methods was the pairwise correlation coefficient. Even with a larger number of deviations (pH 3.0 vs 4.4 formulation) the r^2^ values decreased only slightly. The scaling of the relative deviation resulted in a more sensitive parameter, as shown in Fig. 4b. Amezcua and Szabo described the sensitivity of the correlation approach to structural changes using similar methodology^23^. When performing a reduction of the disulphide bonds of the reference listed drug (RLD) in the Amezcua paper, the r^2^ value of the normalized intensities in the ^1^H-^13^C HSQC spectra dropped to 0.96 for a 6% reduction, 0.88 for a 28% reduction and to 0.60 for a 100% reduction, raising a question about the sensitivity of this approach to small structural changes. The method that compared the signal centre positions in the spectra can be extended to compare the whole spectra.

The PCA method detected the direction of variation for the ^1^H and ^15^N chemical shift data in the high-dimensional chemical shift space. The projection onto the plane of two of the first two eigenvectors was able to separate the NMR samples into three distinct groups, corresponding to the G-CSF at the three different pH values (Fig. 4a). This method was sensitive enough to detect the sample in the pH 3 formulation as an outlier falling outside the Hotteling’s T2 ellipse at the 0.95 confidence level. The two other methods, i.e., the distance metrics and the hierarchical clustering using the complete linkage algorithm, were also sensitive enough to show the differences between the pH values of 3.0, 4.0 and 4.4 and were able to group the NMR samples according to their similarity.

The advantage of the tolerance-interval approach was shown in the analysis of the biosimilar filgrastim and rituximab spectra. When comparing the ^1^H-^15^N HSQC biosimilar-drug and the reference-product spectra at the same pH value, none of the peaks fell outside the tolerance interval determined for the reference-product batches. The method did, however, show differences when the proteins at the two different pH values were compared.

The last method used was the image-difference analysis. In this method the two-dimensional biosimilar spectrum was compared to the spectrum of the reference product by calculating the differences in the normalized signal intensity between the two spectra. The results in Fig. 6 show that the Sandoz biosimilar rituximab product spectra were highly comparable to the reference product, i.e., the spectral-difference score for region 1 was in the range 1.11 to 1.13, which was comparable to the value of 1.13 for the comparison of the two rituximab reference product batches.

Although the described comparability methods could in principle be used for both spectral types, i.e. heteronuclear HSQC and homonuclear NOESY spectra, the scope of use of the mathematically-based comparability metrics depends on the ability to accurately quantify peaks and spectral parameters such as resolution. The HSQC experiments offer much better resolution than NOESY spectra and are not affected by interfering excipient signals. They however lack the sensitivity since they use the naturally occurring ^15^N or ^13^C isotopes. ^1^H-^15^N and ^1^H-^13^C HSQC experiments are the most suitable spectra to compare protein amide and methyl fingerprints. They could be complemented by the homonuclear NOESY in specific cases where the higher order structure should be compared (e.g. sidechains). In this case the similarity evaluation should be adapted to include regions to avoid extensive signal overlap. The low resolution of the NOESY spectra compared to ^1^H-^15^N HSQC would make quantitation of parameters such as image peak centres and linewidths extremely difficult due to high signal overlap in the e.g. aliphatic or amide regions. Unreliable estimates of peak parameters would thus discourage the use of methods such as t-test analogue, PCA, tolerance interval approach and distance metrics for similarity evaluation of NOESY spectra. The methods such as image difference analysis, correlation and spectral overlays would be suitable methods of choice for NOESY spectra. On the other hand the ^1^H-^15^N HSQC and ^1^H-^13^C HSQC spectra could be used with all the methods for biosimilarity evaluation. The special care should be taken to optimize signal the coverage in order to make mathematical evaluation reliable. The consistent results in terms of comparability scores using orthogonal mathematical approaches would increase the reliability of comparability evaluation using similarity metrics.

The framework presented in this paper is meant as a demonstration on how to analyse the NMR spectra, which can be extended to other higher order structure methods. The sensitivity of the data-analysis approaches showed that some methods are much more sensitive than others (e.g., the t-test analogue vs. the correlation method). The aim of these approaches is to introduce objective mathematical metrics instead of qualitative visual spectral comparison and to increase the sensitivity in comparing the higher order structure between two products. The ability to evaluate the degree of similarity between the proteins using the NMR method can serve as an important part of evidence in the process of the biosimilar drug approval by the regulatory agencies.

## Methods

The main experimental challenges encountered were the rapid protein relaxation, the low sensitivity of the ^1^H-^15^N HSQC experiment, the presence of excipients in concentrations that were much higher than that of the protein, the automated signal processing and the objective comparability metrics for the evaluation of the higher-order structure similarity.

### Sample preparation

#### Biosimilar and originator filgrastim samples

An experimental strategy was developed to optimize the sensitivity of the NMR in the drug-product formulation buffer. Experimental conditions, such as temperature, pH and protein concentration, were optimized. In total, 13 samples were prepared for the NMR similarity study of biosimilar and originator filgrastim: 7 samples in the Neupogen formulation buffer, pH 4.0 (originator filgrastim US and EU, biosimilar filgrastim batch 1 (3 replicates), batch 2 and batch 3), 5 samples in the Zarxio formulation buffer, pH 4.4 (originator filgrastim US and EU, biosimilar filgrastim batch 1, batch 2 and batch 3) and a biosimilar filgrastim batch 2 sample in a pH 3.0 buffer. The Neupogen US and EU batches were produced by Amgen and purchased from market in 2012 (purity 99.9% by size exclusion chromatography). The originator filgrastim reference products in the Neupogen formulation (10 mM acetic acid, 50 mg/mL Sorbitol, 0.04 mg/mL Tween 80, pH 4.4) were pooled and concentrated to a final concentration of ≈1 mM using an Amicon Ultra-15 centrifugal device with a cut-off value of 3 kDa. 10% of D_2_O was added to the final solution, followed by a pH adjustment. In the case of the biosimilar filgrastim (Zarxio produced by Sandoz, purity 99.9%) the sample’s drug-product buffer (10 mM glutamic acid, 50 mg/mL Sorbitol, 0.04 mg/mL Tween 80, pH 4.4) was first exchanged with the Neupogen formulation buffer, followed by concentrating to ≈1 mM, addition of 10% D_2_O and a pH adjustment. To test the reversibility of the formulation’s influence on the chemical shifts, both sets of samples, i.e., the biosimilar and originator filgrastim, were prepared in the Zarxio drug-product formulation buffer (10 mM glutamic acid, 50 mg/mL Sorbitol, 0.04 mg/mL Tween 80, pH 4.4).

#### Rituximab - monoclonal antibody

The rituximab monoclonal antibody presented an enormous technical challenge due to its large size (molecular weight ≈144.5 kDa)^29^,^30^. As a result of the large number of hydrogen nuclei the 1D spectra were highly crowded and exhibited an extensive signal overlap. To improve the resolution 2D NMR ^1^H-^1^H NOESY and ^1^H-^15^N HSQC NMR experiments were performed. Experimental conditions such as the buffer, temperature, pH and protein concentration were systematically screened to optimize the NMR spectral information. The screening of the conditions resulted in a 25 mM deuterated acetic acid buffer (acetic acid-d4), 154 mM NaCl with a pH value of 5.0, a temperature of 45 °C, and a protein concentration of 0.75 mM for the full-sized mAb and 1.5 mM for the Fab and Fc fragments.

#### Full-size mAb (originator and biosimilar rituximab) samples

The originator reference product (MabThera produced by Roche and purchased from market, purity 99.3% by size exclusion chromatography) and the biosimilar rituximab samples (produced by Sandoz, purity 99.6%) were first dialyzed against 25 mM deuterated acetic acid (acetic acid-d4) containing 154 mM NaCl, pH 5.0. The dialyzed samples were concentrated to the desired concentration. 10% of the D_2_O was added to the sample solution, followed by a pH adjustment.

#### Fab and Fc samples

Due to the fast T2 relaxation it was not possible to obtain a signal-rich ^1^H-^15^N HSQC spectra of full-sized monoclonal antibodies. Therefore, 150 kDa monoclonal antibody molecules were digested into approximately 50 kDa Fab and Fc fragments using immobilized papain. The Fab and Fc fragments were then isolated from the digestion solution by affinity (Protein A) and size exclusion (SEC) preparative chromatography, as shown in Fig. 7. Finally, the Fab and Fc samples that were isolated in the previous steps were concentrated to the desired value and 10% of the D_2_O was added, followed by the pH adjustment to a value of 5.0.

### NMR spectroscopy

#### Filgrastim samples

^1^H-1D experiment with double-pulse field-gradient spin-echo water presaturation (DPFGSE) was recorded on an Agilent 800-MHz spectrometer equipped with a 5-mm, ^1^H/^13^C/^15^N triple-resonance, cryogenic probe head. A total of 256 scans were used for each 1D spectrum. Multiple pre-saturation frequencies were selected in the 1D spectrum of the biosimilar filgrastim and were subsequently used for the 2D NOESY experiments to suppress the excipient signals, such as sorbitol (molar ratio between the sorbitol and the G-CSF protein was 274:1 after concentrating) and Tween 80 signals^42^. In a similar way the acetic acid signal was suppressed in the originator filgrastim formulation.

Two-dimensional NOESY spectra were acquired with 2048 × 256 data points in the direct and indirect dimensions and spectral widths of 12019 Hz in both dimensions^43^,^44^,^45^. A total of 16 transients were used to achieve a sufficient signal-to-noise ratio. Mixing times of 150 and 250 ms were used in NOESY experiments. Water suppression was achieved using a double pulsed-field gradient spin echo module. The ^1^H-^15^N HSQC spectra were recorded using 1024 × 96 data points and a spectral width of 12019 × 2600 Hz^46^. The low sensitivity of the ^1^H-^15^N HSQC experiments (0.4% of the naturally occurring ^15^N isotope) was compensated by using a long acquisition time of ~32 hours and concentration of the protein samples to achieve a sufficient signal-to-noise level. 100% signal coverage was achieved in the spectra using the naturally occurring ^15^N isotope.

#### Rituximab samples

The 1D and 2D NMR experiments were recorded on the Agilent 800-MHz spectrometer equipped with triple-resonance cryogenic probe head. The 2D rituximab ^1^H-^1^H NOESY spectra were recorded at a temperature of 45 °C, whereas the 2D ^1^H-^15^N HSQC spectra were recorded at 40 °C and 45 °C for the Fab and Fc samples, respectively. Two-dimensional NOESY spectra were acquired with 2048 × 256 data points in the direct and indirect dimensions and spectral widths of 12019 Hz in both dimensions. Mixing times of 150 and 250 ms were used. A total of 16 transients were used to achieve a sufficient signal-to-noise ratio. The ^1^H-^15^N HSQC spectra were recorded using 1024 × 96 data points and a spectral width of 12019 × 2600 Hz. The ^1^H-^13^C gCfHSQC spectrum was recorded at 37 °C using 1024 × 128 data points and a spectral width of 12019 × 28161 Hz.

#### Spectral processing and analysis

The NMR data were processed using NMRPipe software^47^. A resolution enhancement was achieved by the apodization of the free induction decay with a shifted square sine-bell window function. The spectral assignment and the analysis were performed using the Sparky 3.113 software^48^. The spectral assignments for the filgrastim samples were performed using the previously available chemical shift assignment databases using both the chemical shift and pattern matching^12^,^15^,^49^,^50^,^51^,^52^.

In order to automate the NMR signal processing for the image-difference analysis in-house software was developed in Python using a Nmrglue module^53^. The Nmrglue module was used to perform operations, such as Fourier transform, zero filling, applying phase offsets and window functions.

The automated phase was set by maximizing the difference between the positive and negative pixels in low-artefact areas with high signals. The baseline was fitted to data in the regions where no protein signals were present. The ratio of the selected and masked data varied from region to region, depending on the protein signal density. Regions with artefacts (such as water or excipient signals) were excluded from baseline fitting. The amplitude offset was set to minimize the spectral-difference score.

#### Comparability metrics (bioinformatics methods)

The following mathematical methods were used to compare biosimilar to reference product:t-test analogue, principal component analysis (PCA), correlation analysis, tolerance interval approach, distance metrics and image difference analysis using SD-scores. The details of calculations can be found in the supplementary material (Comparability metrics chapter).

## Additional Information

**How to cite this article**: Japelj, B. *et al*. Biosimilar structural comparability assessment by NMR: from small proteins to monoclonal antibodies. *Sci. Rep.* **6**, 32201; doi: 10.1038/srep32201 (2016).

## Supplementary Material

Supplementary Information

## Figures and Tables

**Figure 1:**
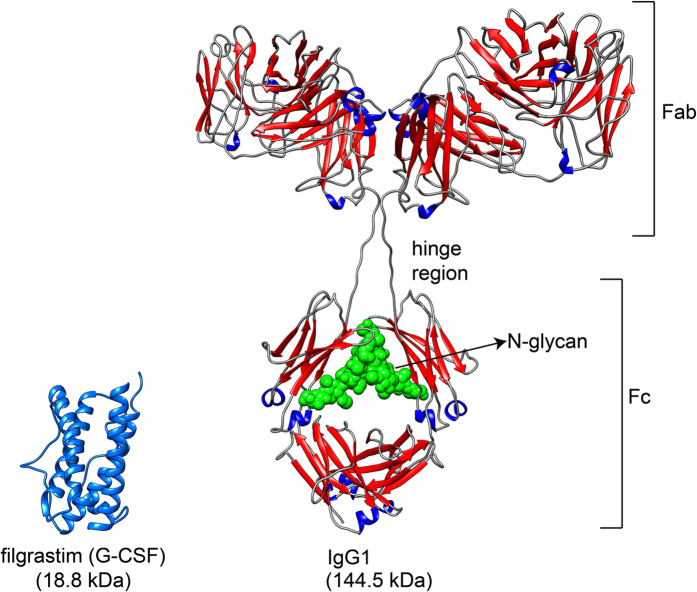
Three-dimensional structure of filgrastim (G-CSF) and IgG1 (e.g. rituximab). Atomic coordinates were taken from the G-CSF NMR structure (PDB ID 1GNC) and theoretical model of IgG1 monoclonal antibody^15^,^54^.
