# Supplementary material for: Biosimilar structural comparability assessment by NMR: from small proteins to monoclonal antibodies
Source: Sci Rep. 2016 Aug 31;6:32201. doi: 10.1038/srep32201 (PMC5006049; doi:10.1038/srep32201)
Supplement: Supplementary Information [file srep32201-s1.pdf]

Supplementary material

Biosimilar structural comparability assessment by NMR: from small proteins to monoclonal antibodies

Boštjan Japelj, Gregor Ilc, Jaka Marušič, Jure Senčar, Drago Kuzman, Janez Plavec

Comparability metrics (bioinformatics methods)

t-test analogue

The t-test analogue is a peak-to-peak comparison method that considers each peak to be an estimate of a resonance position in the spectrum, reflecting the ensemble average conformation. The centre of an individual peak serves as a location estimate, whereas the peak width serves as a variation estimate. The t-test evaluates whether the centre positions of a pair of peaks are statistically different (Supplementary Fig. S1 online). The t-value is defined as the ratio of the distance between the two peak centres (Δ_{AB}) and the sum of the estimates of the peak variances. The Euclidean distance between the centres of samples A and B was measured as:

$$\Delta_{AB} = \sqrt{(\omega_1^A - \omega_1^B)^2 + (\omega_2^A - \omega_2^B)^2} \qquad (1)$$

The variability of the peak position in samples A and B (sd_{A}, sd_{B}) was approximated by the peak width at half of the peak height ($lw^{A}/2$, $lw^{B}/2$), resulting in sd_{AB} as an estimate for the sum of the dispersion of both peaks:

$$sd_A = \sqrt{\left(\frac{lw_1^A}{2}\right)^2 + \left(\frac{lw_2^A}{2}\right)^2},\ sd_B = \sqrt{\left(\frac{lw_1^B}{2}\right)^2 + \left(\frac{lw_2^B}{2}\right)^2} \qquad (2)$$

$$sd_{AB} = \sqrt{{sd_A}^2 + {sd_B}^2} \qquad (3)$$

The ratio between (Δ_{AB}) and sd_{AB} is defined as:

$$t_{AB}{}^{*} = \frac{\Delta_{AB}}{sd_{AB}} \qquad (4)$$

Note that $t_{AB}{}^{*}$ is not equal to the t parameter since in the standard t-test the sample variability depends on the standard deviations of both groups and the number of members of the group. The value $t_{AB}{}^{*}$ was a basis to test the significance of the chemical shifts. The risk level (called the alpha level) was set to 0.05. Benjamini-Hochberg correction for controlling the false discovery rate was used to overcome the problem of multiple comparisons[1, 2]. Other methods were also tested, including the Sidak, Hochberg's, Scheffe's method, the Dunnet test, the Tukey test and the Games-Howell test[3].

The shifts and their significance were calculated in the R programming language for all backbone peaks of the pairwise compared samples. The results were exported to Spotfire 3.3.2 (TIBCO) for visualization.

PCA

Principal component analysis (PCA) is a statistical, unsupervised learning method whose aim is to reduce the dimensionality of multivariate data sets by preserving as much of the relevant information as possible[4, 5].

PCA models were calculated using Simca 13 software (Umetrics). Principal component analysis (PCA) was performed on the ^{1}H-^{15}N HSQC spectra of 13 observations using 162 ^{1}H and ^{15}N chemical shifts for the individual residues as x-variables.

Correlation analysis

The pairwise similarity between the spectra was measured using the Pearson correlation coefficient (r). This coefficient measures the degree and direction of the linearity between two vectors **x** and **y**[6]. The chemical shifts from the ^{1}H-^{15}N HSQC spectra were extracted as vectors,

which were in turn correlated. A slightly different approach was used before to quantitatively compare the FT-IR and NOESY spectra[7, 8, 9]. The correlation coefficient was calculated for the weighted ^1H and ^{15}N chemical shifts according to the formula:

$$\delta_w = \sqrt{\frac{1}{N}\sum_{i=1}^{N}(\alpha_i \delta_i)^2}, \qquad (5)$$

where α_i is a scaling factor to compensate for the difference in the ^1H and ^{15}N ranges[10]. The calculations and figures preparation were made in R programing language. The r values were normalized in such a way that the difference between the EU and US reference product was normalized to a value of 1 and other r values in the correlation matrix were multiplied by the reference product scaling factor.

Tolerance interval approach

The quantitative biosimilarity evaluation using the tolerance-interval approach is based on an assumption that the spectra of the biosimilar sample can differ from the reference-product spectra only to extent that the spectra of different batches of the reference product differ from each other. A peak is significantly shifted if the distance exceeds the tolerance limit at a predefined confidence level (1-α) and proportion of the population (P).

Each detected peak in the Fab and Fc peak in the ^1H-^{15}N HSQC spectrum was described by its label, the ^1H peak chemical shift (ω_1) and the ^{15}N chemical shift (ω_2) and the peak height and the signal-to-noise (S/N) ratio. The spectra were pairwise compared (e.g., Fab originator vs Fab biosimilar rituximab drug product batch 1) by analysing the magnitude of the peak shifts. The statistical evaluation of the results was performed for the ^1H and ^{15}N dimensions separately, as well as for the weighted chemical shifts, which were calculated using the equation:

$$\Delta_{AB,w} = \sqrt{(\omega_{HN}^{A} - \omega_{HN}^{B})^2 + \alpha^2(\omega_{N}^{A} - \omega_{N}^{B})^2} \quad (6)$$

where $\Delta_{AB,w}$ is the weighted Euclidean distance between the peak centres of samples A and B, with ω_{HN}^{A} and ω_{HN}^{B} being the sample A and B peak centres in the ^{1}H, and ω_{N}^{A} and ω_{N}^{B} in ^{15}N dimension, respectively, and α is a scaling factor to compensate for the difference in the, ^{1}H and ^{15}N ranges[10]. Peaks with a S/N ratio ≥ 3 were used for the evaluation. An analysis of the histograms showed that the distribution of the chemical shift differences in the ^{1}H and ^{15}N dimensions, separately, was Cauchy (Lorentzian), rather than normal, which was not able to sufficiently describe the positive excess kurtosis (leptokurtic distribution). The distribution of weighted chemical shifts was approximated using a Gamma distribution. The tolerance intervals were calculated using a script written in the R programming language. The R-package "tolerance" was used to calculate the tolerance intervals[11]. The confidence level was set to 0.95, whereas P was set to 0.99 for the normal and Gamma distributions, and 0.9 for the Cauchy distribution, which ensured that all the reference-product shifts were within the tolerance limits. The method was sensitive enough to reflect changes in the formulation of the 0.4 and 1.0 pH unit.

Euclidean distance

The similarity between spectra as objects can be measured using the distance metric. The common distance is the Euclidean, a special case of Minkowski distance, which is defined as:

$$d(\vec{p},\vec{q}) = \sqrt{(q_1 - p_1)^2 + (q_2 - p_2)^2 + \dots + (q_n - p_n)^2} = \sqrt{\sum_{i=1}^{n}(q_i - p_i)^2}, \quad (7)$$

where $\vec{p} = (p_1, p_2, \dots, p_n)$ and $\vec{q} = (q_1, q_2, \dots, q_n)$ are two vectors in the Euclidean *n*-space. The distances between the samples were calculated using a custom-written R-script, which was also used for a visualization of the results.

Hierarchical cluster analysis

An agglomerative hierarchical cluster analysis was used to group the ^{1}H and ^{15}N HSQC spectral spectra shifts into clusters with the goal to find the similarity between the samples[12]. The complete linkage algorithm was used as part of an R script to calculate the clusters[12]. The algorithm first assigned each object to its own cluster, followed by joining the two most similar by the shortest distance clusters until only a single cluster remained at the end.

Image-difference analysis

The difference between two spectra can be used to evaluate the similarity of two molecules. If the recorded molecules are the same, the difference spectrum should be blank.

The spectra were processed by first defining the regions of interest, which included parts of the NOESY spectra where only protein signals were present. The spectra were then processed using automated phase correction and baseline subtraction, followed by a calculation of the normalized volume of the squared signal in the subtracted spectra. After the subtraction, the spectral-difference score ($SD\text{-}score_i$) was calculated as the square root of the ratio between the average squared signal intensity I in the selected region, Ω_i, and the average squared signal intensity I in the noise region, Ω_0, according to the equation bellow:

$$SD\text{-}score_i = \sqrt{\frac{1}{S_i}\sum_{x,y\,\in\,\Omega_i} {I_{x,y}}^2 \Big/ \frac{1}{S_0}\sum_{x,y\,\in\,\Omega_0} {I_{x,y}}^2} \qquad (8)$$

The area S describes the number of pixels (x, y pairs) in respective region. The noise was calculated in the 10-12 ppm region where none of the protein signals were observed. If the recording molecules were the same and the artefacts were absent, the $SD\text{-}score_i$ value would ideally have a value of 1. The

residual signals in the difference spectrum indicate directly the sample differences arising from the protein structure.

Sensitivity of the NMR method to small pH changes

^{1}H and ^{15}N chemical shifts measure the locally induced magnetic field; therefore, the ^{1}H-^{15}N HSQC fingerprint spectra are highly sensitive to local protein conformation and the environment such as pH and ionic strength[13-15]. Chemical shifts are predominantly intra-molecular interactions, but they also have a significant intermolecular component (e.g. the chemical shifts are slightly different in different solvents (ionic strength, pH)). During the study the influence of the pH shift on the ^{1}H and ^{15}N resonance positions was observed when the pH was increased from 4.0 to 4.4 in the Neupogen® and Zarxio® drug product formulations, respectively. In order to systematically demonstrate the sensitivity of the method to pH changes, a biosimilar protein was prepared in three different formulations with pH values of 3.0, 4.0 and 4.4 (Supplementary Fig. S2 online). The pH-induced chemical shifts were observed and modelled before by Pujato[14]. The most prominent shifts in the ^{1}H-^{15}N plane were observed for the peaks V48, G73, S76, S80, L103, D104, A127, S142 and L161 (Supplementary Fig. S2 online), which confirmed the formulation-pH influence on the protein conformation. In addition, the sensitivity of the HSQC spectra was additionally confirmed by observing dynamic conformational changes such as the proline cis-trans isomerization, resulting in satellite peaks (e.g. G4').

References:

1. Benjamini, Y., Hochberg, Y. Controlling the false discovery rate: a practical and powerful approach to multiple testing. *J. R. Stat. Soc. Ser. B*, **57**, 289–300 (1995).

2. Benjamini, Y., Yekutieli, D. The control of the false discovery rate in multiple testing under dependency. *Ann. Stat.* **29**, 1165–1188 (2001).

3. Nichols, T. & Hayasaka, S. Controlling the familywise error rate in functional neuroimaging: a comparative review. *Stat. Methods in Med. Res.* **12**, 419-446 (2003).

4. Joliffe, I.T. *Principal Component Analysis.*, Edn. 2nd. (Springer-Verlag, New York; 2002).

5. Pearson, K. LIII. On lines and planes of closest fit to systems of points in space. *Philos. Mag. Ser.* **6**(2), 559-572 (1901).

6. Becker, R.A., Chambers, J. M. and Wilks, A.R. *The New S Language.* (Chapman and Hall/CRC, 1988).

7. Amezcua, C.A., Szabo, C.M. Assessment of higher order structure comparability in therapeutic proteins using nuclear magnetic resonance spectroscopy. *J. Pharm. Sci.* **102**, 1724-1733 (2013).

8. Prestrelski, S.J., Tedeschi, N., Arakawa, T. & Carpenter, J.F. Dehydration-induced conformational transitions in proteins and their inhibition by stabilizers. *Biophys.J* . **65**, 661-671 (1993).

9. Meyer, N.H., Zangger, K. Enhancing the resolution of multi-dimensional heteronuclear NMR spectra of intrinsically disordered proteins by homonuclear broadband decoupling. *Chem. Commun.* (Cambridge, England) **50**, 1488-1490 (2014).

10. Williamson, M.P. Using chemical shift perturbation to characterise ligand binding. *Prog. Nucl. Magn. Reson. Spectrosc.* **73**, 1-16 (2013).

11. Young, D.S. tolerance: An R package for estimating tolerance intervals. *J. Stat. Softw.* **36**, 1-39 (2010).

12. Everitt, B. *Cluster Analysis.*, Edn. 5th. (Wiley, Chichester, West Sussex, U.K.; 2011).

13. Pujato, M., Bracken, C., Mancusso, R., Cataldi, M. & Tasayco, M.L. pH dependence of amide chemical shifts in natively disordered polypeptides detects medium-range interactions with ionizable residues. *Biophys.J.* **89**, 3293-3302 (2005).

14. Levit, M., H. *Spin Dynamics: Basics of Nuclear Magnetic Resonance.* Edn. 2nd. (John Wiley and Sons, Chichester, U.K.; 2008).

15. Tadeo, X., Pons, M. & Millet, O. Influence of the Hofmeister anions on protein stability as studied by thermal denaturation and chemical shift perturbation. *Biochemistry 46*, 917-923 (2007).

SUPPLEMENTARY FIGURE LEGENDS:

Supplementary figure S1. t-test analogue peak-shift calculation

Supplementary figure S2. Overlay of the Zarxio® drug product batch 2 G-CSF spectra in three different formulations –pH 3.0 (blue), pH 4.0 (red) and pH 4.4 (orange).

Supplementary figure S3. Statistical evaluation of the biosimilar and originator rituximab Fab spectra. (a) Comparison between biosimilar product biosimilar rituximab Fab fragment (green) with two originator Fab fragment spectra (orange and cyan for originator batch 1 and 2 respectively). (b) The effect of pH change on the ^{1}H-^{15}N HSQC spectral shifts of the biosimilar rituximab Fab fragment at pH 4.6 (purple), 5.0 (yellow) and 5.4 (magenta). The histograms in (c) and (d) show the sensitivity of the comparison for two originator rituximab batches and the biosimilar to the originator rituximab in the pH 5.0 formulation. Panels (e) and (f) show the sensitivity of the tolerance-interval approach to the pH changes from 5.0 to 4.6 and 5.0 to 5.4, respectively.

Supplementary figure S4. ^{1}H-^{13}C HSQC spectrum of the biosimilar rituximab Fab at pH 6.5 recorded at 37 °C.

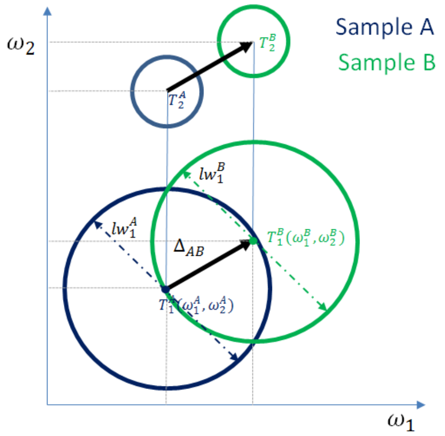

Supplementary figure S1.

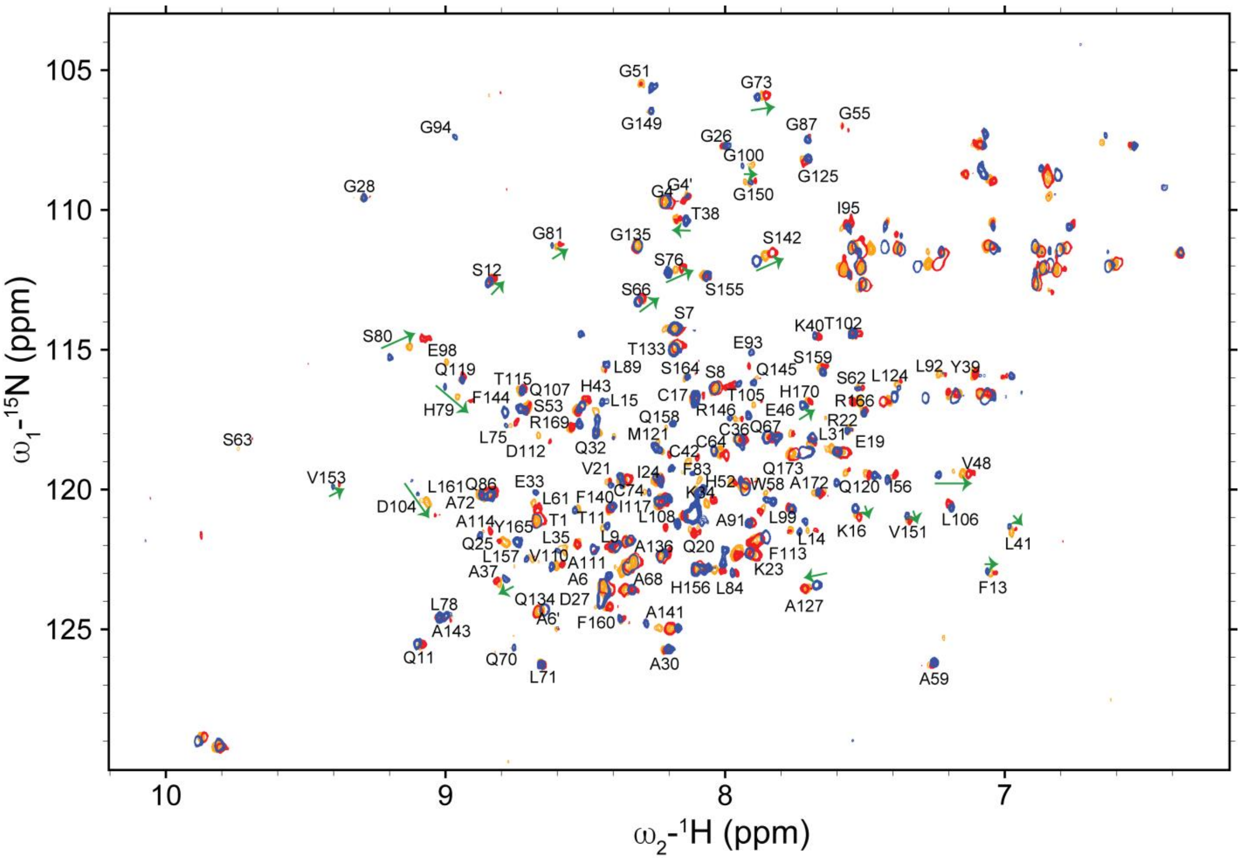

Supplementary figure S2.

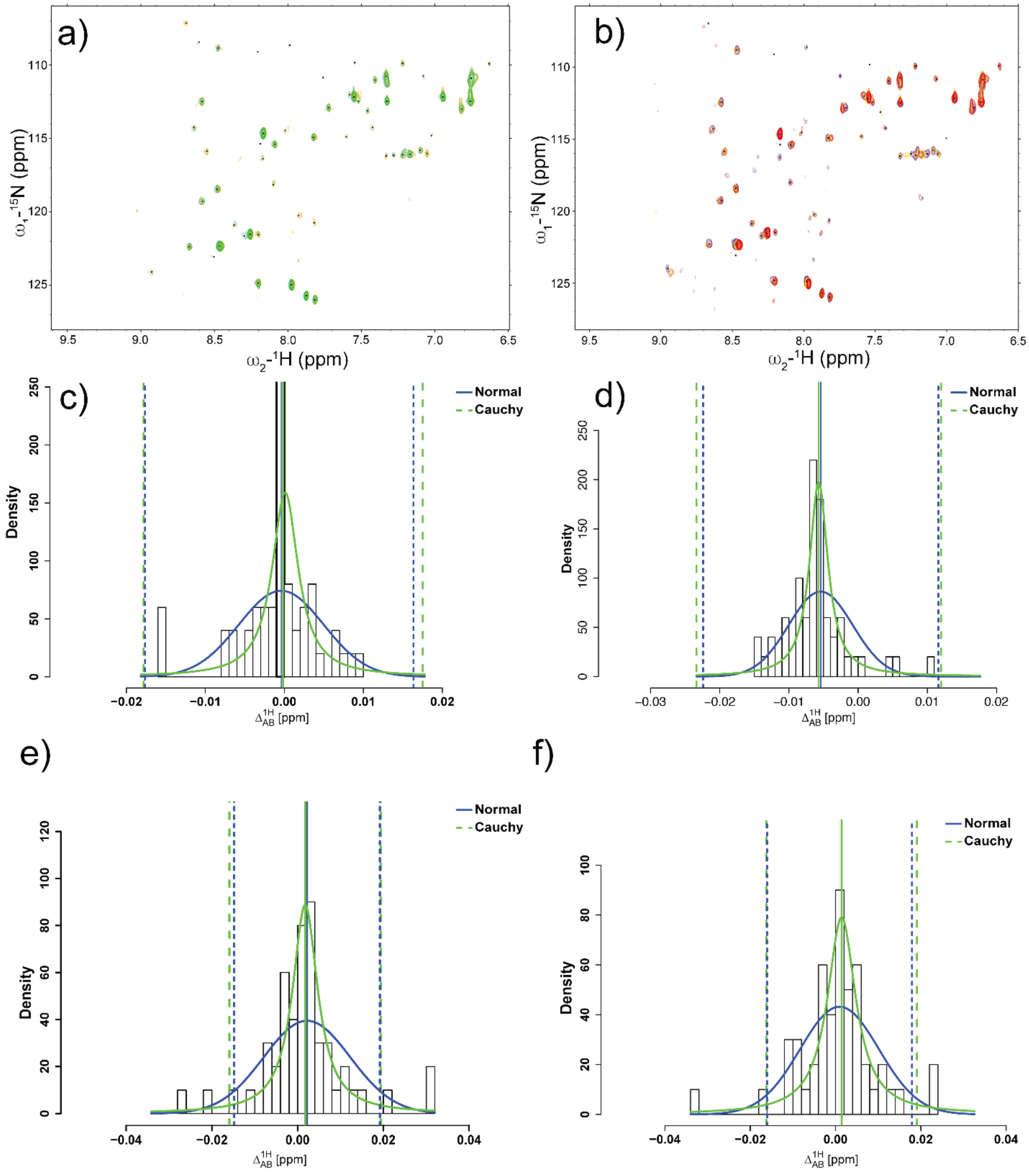

Supplementary figure S3.

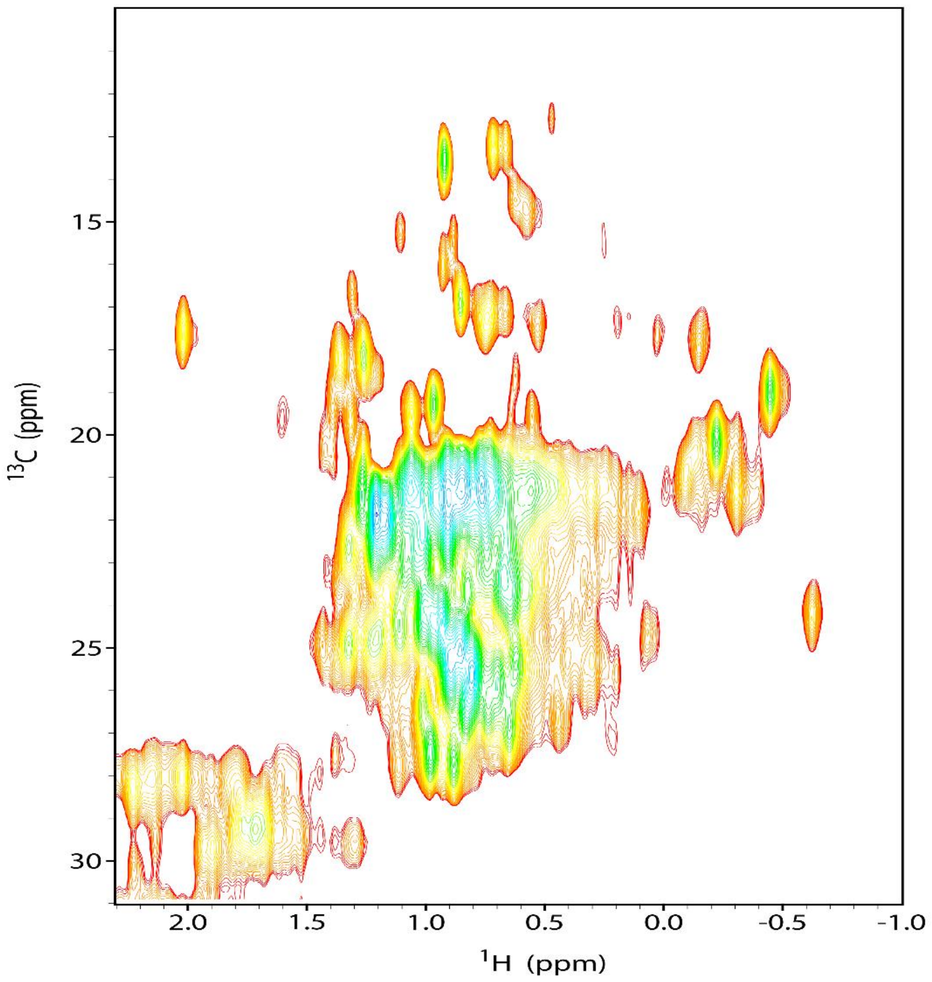

Supplementary figure S4.
